# Demographic and clinical correlates of discordant QuantiFERON TB Gold tuberculosis screening results in a low-incidence setting

**DOI:** 10.1128/spectrum.02822-25

**Published:** 2026-02-09

**Authors:** Gaurav K. Sharma, Farah Haq, Arthur H. Totten, Luis A. Marcos, Charles Kyriakos Vorkas

**Affiliations:** 1 Division of Infectious Diseases, Department of Medicine, Renaissance School of Medicine at Stony Brook University 12300, Stony Brook, New York, USA; 2 Division of Clinical Preventive Medicine, Department of Family, Population and Preventive Medicine, Renaissance School of Medicine at Stony Brook University 12300, Stony Brook, New York, USA; 3 Department of Pathology, Stony Brook University Hospital 200793, Stony Brook, New York, USA; 4 Department of Microbiology and Immunology, Renaissance School of Medicine at Stony Brook University 12300, Stony Brook, New York, USA; 5 Center for Infectious Diseases, Renaissance School of Medicine at Stony Brook University 12300, Stony Brook, New York, USA; Icahn School of Medicine at Mount Sinai, New York, New York, USA

**Keywords:** *Mycobacterium tuberculosis*, tuberculosis, low-incidence setting, serial testing, QuantiFERON-TB Gold Plus, interferon-gamma release assay, latent tuberculosis infection

## Abstract

Interferon-γ release assays (IGRAs), such as the QuantiFERON-TB Gold Plus (QFTTB), are commonly used to detect past exposure to *Mycobacterium tuberculosis* complex (*Mtb*), the cause of tuberculosis (TB). IGRA-positive (IGRA+) asymptomatic individuals are diagnosed with presumptive latent tuberculosis infection (LTBI) and often offered therapy to prevent active disease. However, discordant results during serial testing pose challenges for interpretation and may lead to unnecessary treatment. We conducted a retrospective study of subjects who received QFTTB testing at Stony Brook Medicine between October 2020 and March 2024 to identify sociodemographic and clinical variables associated with quantitative QFTTB results. A total of 743 subjects were analyzed, including all 436 QFTTB-positive (QFTTB+) cases of 11,641 tests ordered (3.7%), of whom 16 were diagnosed with active TB during the 4-year study period within the context of a reported incidence rate of 3.4/100,000 persons per year in Suffolk County, a region of low TB incidence. A random sample of 307 age-sex-matched QFTTB-negative controls was included. Of 203 subjects undergoing serial QFTTB testing, 170 (83.7%) had concordant results, while 33 (16.3%) showed discordance—23 (69.7%) with reversion and 10 (30.3%) with conversion. Conversions occurred in significantly older subjects (mean age 51.1 ± 15.0 vs 37.0 ± 15.6, *P* = 0.025) and over longer intervals (415.1 vs 91.2 days, *P* = 0.026). Our findings reinforce the use of confirmatory or repeat testing before initiating LTBI therapy, particularly when testing intervals are short (<6 months) or results fall near the diagnostic cutoff (0.35 IU/mL).

**IMPORTANCE:**

Reliable interpretation of interferon-γ release assays (IGRAs) is critical for the diagnosis and management of latent tuberculosis infection (LTBI). However, variability in test performance during serial or confirmatory testing complicates clinical decision-making and may result in unnecessary treatment. Our study demonstrates that demographic factors, clinical comorbidities, and testing intervals contribute to discordant QuantiFERON-TB Gold Plus results. These findings underscore the need to integrate epidemiologic risk, pre-test probability of *Mycobacterium tuberculosis* complex exposure, clinical history, and repeat testing when appropriate before initiating LTBI therapy. Improved understanding of IGRA variability can strengthen both patient care and research applications, including tuberculosis vaccine and protective biomarker studies.

## INTRODUCTION

Tuberculosis (TB) is a significant public health challenge, with an estimated 10.8 million new cases and 1.25 million deaths annually worldwide (1). A cornerstone of TB control efforts is early detection of *Mycobacterium tuberculosis* complex (*Mtb*) exposure in asymptomatic individuals using immunodiagnostic screening and treatment of presumed latent tuberculosis infection (LTBI). This intervention can reduce the 10% lifetime risk of progression to active TB and mitigate potential transmission to naïve contacts (2). Accurate screening for LTBI is essential for public health strategies aimed at TB prevention with the goal of elimination of *Mtb* in human reservoirs.

Interferon-γ release assays (IGRAs), such as the QuantiFERON-TB Gold Plus (QFTTB) and T-SPOT.TB, have emerged as alternatives to the traditional tuberculin skin test (TST) for diagnosis of LTBI, with guidelines favoring IGRA testing over TST for TB screening (3). These blood-based assays measure the release of interferon-γ (IFN-γ), a cytokine secreted by T lymphocytes upon recognition of *Mtb* antigens, offering improved specificity over TST by minimizing cross-reactivity with Bacillus Calmette-Guérin (BCG) vaccination and non-tuberculous mycobacteria (4). QFTTB is an IGRA that utilizes two antigen tubes—TB1 and TB2—each containing synthetic peptide pools derived from early secreted antigenic target-6 and culture filtrate protein-10, which are absent from BCG strains and most non-tuberculous mycobacteria (4, 5). While the TB1 tube is optimized to stimulate CD4^+^ T-helper cells restricted by major histocompatibility complex class II (MHC II), responses may also be elicited from CD8^+^ T-cells restricted by MHC I (6). The TB2 tube includes additional shorter peptide pools designed to enhance sensitivity by stimulation of both CD4^+^ and CD8^+^ T cells (6, 7). The assay includes a mitogen tube as a positive control and a NIL tube to measure basal IFN-γ levels. Interpretation is based on pre-determined “IFN-γ minus NIL” cutoffs measured by enzyme-linked immunosorbent assay of whole blood (8). If the mitogen tube “IFN-γ minus NIL” concentration does not reach the positive control threshold, the result is reported as “indeterminate” and the antigen tubes are not tested. This assay has been widely incorporated into TB screening programs in both high- and low-incidence settings and is used as a primary measure of efficacy in experimental preventive TB vaccine clinical trials (4, 9, 10). For example, in one Phase III clinical trial, IGRA conversion was used to measure incident primary infection in otherwise baseline asymptomatic IGRA-negative individuals in high-risk settings (10).

Despite its utility for assessing past *Mtb* exposure, serial IGRA testing commonly demonstrates discordant results that can confound accurate interpretation of *Mtb*-specific immune responses (11, 12). Within-subject assay performance raises concerns about reproducibility and reliability as a screening test, particularly in regions of low incidence (13, 14). For example, a person may test negative and subsequently positive with no clear evidence of recent *Mtb* exposure (11, 12, 14). In turn, confirmatory IGRA testing may yield a discordant negative result. Discordant results may misclassify individuals, leading to missed opportunities to initiate LTBI therapy or inappropriate antibiotic prescriptions (12, 15, 16). Moreover, discordant QFTTB testing as a measure of efficacy in clinical trials may confound the development of new TB vaccines (10).

Multiple factors have been reported to contribute to discordant IGRA results, including a dynamic host immune response, assay cutoff thresholds, and specimen processing (17, 18). Importantly, 8–19% of microbiologically confirmed active TB cases have negative IGRA results that are attributed to T-cell exhaustion during chronic infection and reflect inherent limitations in using TB immunodiagnostics to diagnose active disease (17). It has also been reported that individuals with recent viral infections, malnutrition, or chronic inflammatory diseases may experience periods of immunosuppression, potentially leading to false-negative results (17, 19). Assay-specific variability has also been documented, with studies showing that different IGRA platforms (QFTTB vs T-SPOT.TB) may yield conflicting results in the same individual (20).

Our study examined the hypothesis that sociodemographic and clinical variables correlate with QFTTB results, focusing on discordant serial testing in a community-based population in a low-incidence setting in Suffolk County, Long Island, NY that reports 3.4 cases/100,000 persons per year (21). This classifies Suffolk County as a low-incidence region using the WHO definition of <10 cases per 100,000 persons per year (22). We found that discordant QFTTB results were common during serial testing in a low-incidence region. Younger patients with short retesting intervals showed high reversion rates, while converters were older and had more chronic illnesses and medication use. These patterns demonstrate frequent biological and operational variation that affects accurate assessment of TB risk in low-incidence settings.

## MATERIALS AND METHODS

This was a retrospective electronic medical record (EMR) review of human subjects who underwent routine standard of care QFTTB testing (QIAGEN, Germantown, MD, USA) at Stony Brook Medicine between 6 October 2020 and 20 March 2024, based on the available institutional data extraction window. This study was approved by the Stony Brook Medicine institutional review board (IRB2024-00149; PI: Vorkas CK) and informed consent requirements were waived. All extracted data were de-identified to ensure subject privacy and confidentiality. Subjects were identified through laboratory records of completed QFTTB testing. All individuals with quantitative TB1 and TB2 results were eligible for inclusion. Subjects were excluded if they had incomplete QFTTB data (e.g., missing TB1, TB2, or overall result) or if QFTTB resulted as indeterminate. For those who underwent serial testing, discordance was defined as either conversion (negative-to-positive result) or reversion (positive-to-negative result).

Demographic and clinical characteristics were examined, including age, sex, race/ethnicity, and medical comorbidities such as infection status with human immunodeficiency virus (HIV), cardiovascular disease, autoimmune disease, diabetes, chronic obstructive pulmonary disease (COPD), chronic kidney disease, and cirrhosis. Medication history was also recorded, including immunosuppressive therapy (e.g., tumor necrosis factor-α inhibitors and corticosteroids), cardiovascular medications, and pain management drugs. QFTTB results included dates of testing, quantitative TB1 and TB2 antigen responses, and categorical test results (positive or negative). Quantitative IFN-γ levels were reported in international units per milliliter (IU/mL).

Sociodemographic and clinical correlates of IFN-γ responses were first analyzed in the total study population, and then in subjects who underwent serial testing stratified by “concordant” and “discordant” results. Descriptive statistics were used to summarize continuous variables (e.g., TB1/TB2 levels) and are reported as means with standard deviations (SDs) and compared using Welch’s *t*-tests. Categorical variables (e.g., QFTTB qualitative test result, comorbidities, and discordance type) were summarized as frequencies and analyzed using chi-square or Fisher’s exact tests. Nil, Mitogen, TB1, and TB2 results were correlated with age, sex, race/ethnicity, comorbidities, TB exposure history, TB treatment status, medications, and duration between testing using univariate linear regression analyses. Quantitative changes in TB1 and TB2 values were calculated by subtracting baseline from follow-up values. Time intervals between serial tests were calculated in days.

Discordance analysis of the change in TB1 and TB2 values compared converters and reverters using paired *t*-tests and Wilcoxon signed-rank tests, depending on data distribution. One-way ANOVA was used for comparisons across more than two groups when appropriate. The impact of comorbidities and medication classes on changes in NIL, TB1, TB2, and Mitogen values was assessed using two-way ANOVA, to evaluate both the main effects (discordance group and comorbidity/medication type) and their interaction. The association between discordance type and specific comorbidities (e.g., HIV, cardiovascular disease, and autoimmunity) was evaluated using chi-square tests. Serial TB1 and TB2 test values were assessed using linear regression models.

Statistical analysis and data visualization were conducted using RStudio (version 2024.04.2+764; RStudio, PBC, Boston, MA, USA). Statistical significance was assessed using thresholds of *P* < 0.05 and *P* < 0.01, as indicated.

No *a priori* power calculation was conducted given the retrospective nature of this study. However, the inclusion of all QFTTB-positive cases (*n* = 436) over a 4-year window, along with a matched sample of negative controls, maximized the statistical power for detecting differences in rare outcomes, such as test discordance. Based on the final sample size of 743 subjects, the study had >80% power (*α* = 0.05) to detect medium effect sizes (Cohen’s *d* ≈ 0.5) in TB1 and TB2 responses between discordance groups. The study period was selected to ensure capture of serial QFTTB testing and representative comorbidities for the subgroup analyses.

## RESULTS

### Study population

Out of 11,641 QFTTB tests ordered between 6 October 2020 and 20 March 2024, a total of 436 (3.7%) cases tested positive, and a random age- and sex-matched convenience sample of 307 QFTTB-negative controls was selected from a pool of 10,959 negative QFTTB results. An additional 243 cases were excluded from the analysis because they did not appropriately respond to the mitogen-positive control and TB1/TB2 testing was not performed (“indeterminate” QFTTB results). A total of 743 subjects were included in the study. QFTTB testing indications most commonly included routine risk-stratification for *Mtb* exposure and progression to active TB disease in healthcare settings: employee health screening, including Veterans Home staff (50.8%), followed by renal transplant preoperative evaluation (12.2%), emergency department visits with symptoms (9.1%) or without symptoms (8.8%), obstetric evaluations (3.9%), and the remaining 15.2% during routine clinical care including HIV, rheumatology, outpatient infectious disease, and other advanced specialty clinics. The mean age was 47.0 years (SD ± 16.5), with 40.9% identifying as male and 59.1% as female. The racial/ethnic composition included 47.97% White, 8.94% Asian, 20.46% Black/African American, 22.09% Hispanic/Latino, and 0.54% American Indian or Alaskan Native (Table 1).

**TABLE 1:** Sociodemographic and clinical characteristics of the study population^*a*^

Characteristic	Value	
Total Number of Participants	743	
Mean Age (SD)	47.0 years (16.5)	
Gender	40.9% Male, 59.1% Female	
Racial/Ethnic Composition	White	47.97%
	Asian	8.94%
	Black/African American	20.46%
	Hispanic/Latino	22.09%
	Indian/Alaskan Native	0.54%
QFTTB Test Result	436 Positive, 307 Negative	
Latent TB	420 cases	
Active TB	16 cases	
Discordant Results	33 cases	
Comorbidities		
Cardiovascular Disease	29.4%	
Cancer	12.5%	
Chronic Obstructive Pulmonary Disease (COPD)	9.2%	
HIV	6.5%	
Hepatitis	3.0%	

^*a*^ Sampling method: This data set represents QFTTB tests conducted at Stony Brook Medicine from 6 October 2020 to 20 March 2024, where all positive cases (*n* = 436) were included in the analysis and compared to a convenience sample of 307 age- and sex-matched QFTTB-negative controls. All QFTTB testing performed in these subjects during the study period was included in the analysis.

Of the QFTTB+ cases, 16 (3.67%) were diagnosed with microbiologically confirmed active TB infection, and the remaining 420 (96.33%) subjects were diagnosed with asymptomatic, presumed LTBI. Seventy-five percent of active TB cases were Hispanic immigrants from Central and South America. Two hundred three subjects had serial QFTTB testing, of which 33 (16.2%) demonstrated discordant results. Among all subjects, 29.4% had cardiovascular disease, making it the most prevalent comorbidity in this cohort. Cancer was diagnosed in 12.5% of subjects, 9.2% had COPD, 6.5% were living with HIV, and 3.0% had viral hepatitis (hepatitis B or C viruses) (Table 1).

Among all study subjects, no significant correlations were observed between TB1-NIL, TB2-NIL, Mitogen-NIL, or NIL values and sociodemographic or clinical variables (Fig. S1).

### Concordant versus discordant IGRA testing

A total of 170 (83.74%) individuals had concordant QFTTB results upon serial testing—89 with persistently negative results and 81 with persistently positive results. Thirty-three subjects (16.26%) had discordant results. The concordant group included 87 females and 83 males; the discordant group included 17 females and 16 males.

**Fig 1:**
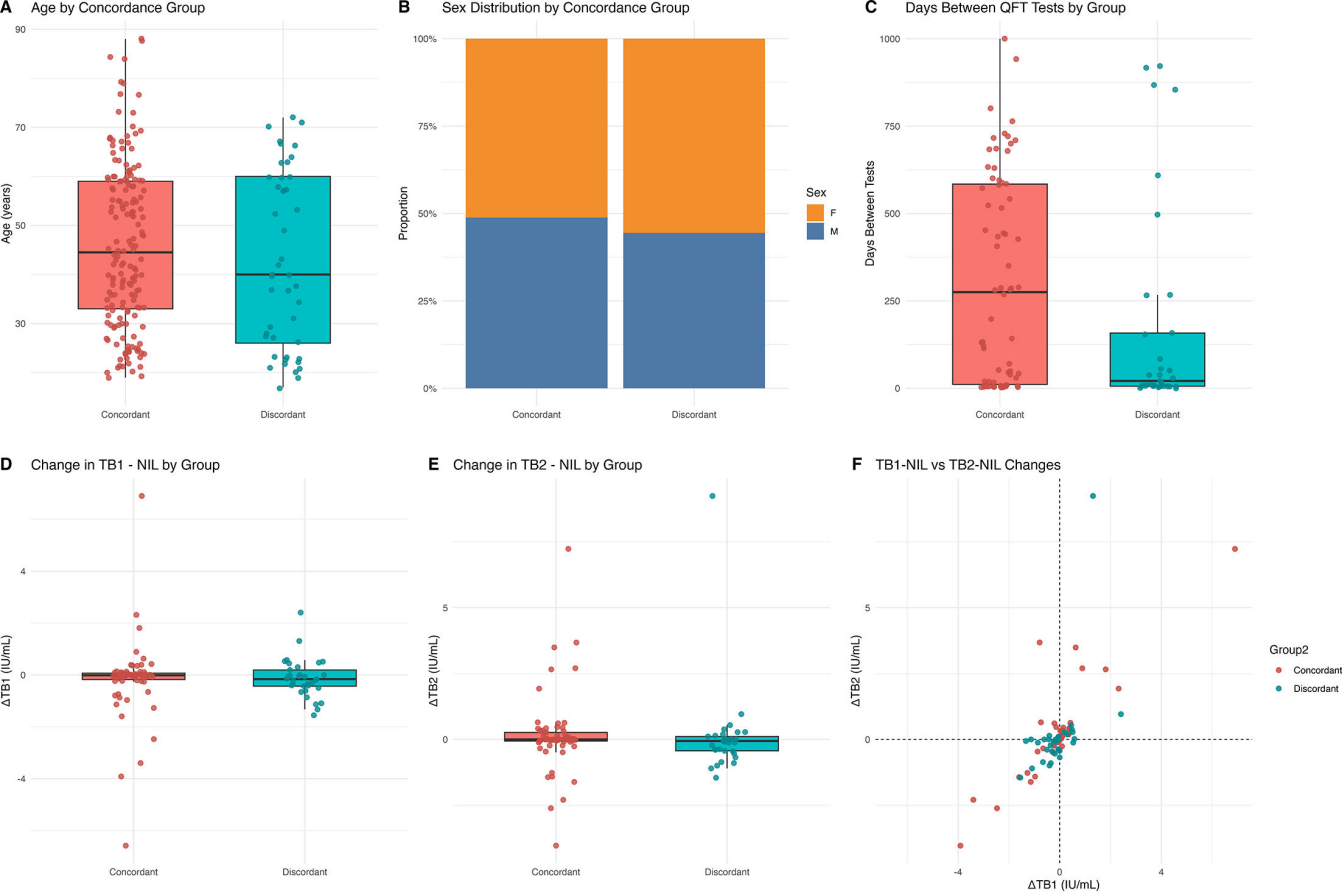
Shared sociodemographic characteristics between serial quantitative QFTTB concordance and discordance groups. (**A**) Age distribution by concordance group, presented as boxplots. Boxes represent the IQR, horizontal lines denote the median, and whiskers extend to 1.5 × IQR (Welch’s *t*-test *P* = 0.288). (**B**) Sex distribution by concordance group (concordant vs discordant), shown as a stacked bar plot. (**C**) Time in days between serial QFTTB tests by group, shown as boxplots with overlaid individual data points (Welch’s *t*-test *P* = 0.085). (**D**) Changes in TB1-NIL IFN-γ response by group, displayed as boxplots with jittered individual data points (*P* = 0.683). (**E**) Changes in TB2-NIL IFN-γ response by group, also shown as boxplots with jittered points (*P* = 0.980). (**F**) Scatterplot of TB1-NIL versus TB2-NIL changes illustrate overlapping distributions across groups. Statistical comparisons were performed using Welch’s *t*-tests. No comparisons reached statistical significance.

There were no statistically significant sociodemographic differences in QFTTB quantitative results between the concordant and discordant serial testing groups (Fig. 1). The mean age of individuals with concordant results was 45.8 ± 20.0 years, compared to 41.3 ± 16.5 years among discordant subjects (*P* = 0.2881; 95% CI: –1.87 to 10.80) (Fig. 1A). Sex distribution did not significantly differ between groups (*P* > 0.05) (Fig. 1B). The average time between serial QFTTB tests did not significantly differ (*P* = 0.085) (Fig. 1C). The median interval between serial tests was 137.5 days (interquartile range [IQR]: 6.75–582.75) for concordant and 28 days (IQR: 6–158) for discordant results (Fig. 1C). No significant differences were observed in TB1 or TB2 changes between concordant and discordant groups (TB1: *P* = 0.6828; TB2: *P* = 0.9799) (Fig. 1D and E). Scatterplot analysis of IFN-γ concentration changes (TB1 vs TB2) revealed overlapping patterns between groups (Fig. 1F). Across all positive tests, TB1 values showed a median of 1.25 IU/mL (IQR: 0.53–3.76) with an average of 2.64, and TB2 values showed a median of 1.22 IU/mL (IQR: 0.51–3.72) with an average of 2.68.

### Discordant IGRA testing

Of the 33 study subjects with discordant results, 23 (69.7%) experienced reversion (positive to negative), while 10 (30.3%) experienced conversion (negative to positive). Five (15.2%) of these discordant cases received LTBI treatment with rifampin or isoniazid/rifapentine at the time of testing; three in the conversion group and two in the reversion group. Among converters, the values associated with the new positive result demonstrated a TB1 median of 0.53 IU/mL (IQR: 0.41–0.73) with an average of 0.754, and a TB2 median of 0.41 IU/mL (IQR: 0.255–0.6275) with an average of 0.509. Among reverters, the values associated with the initial positive result demonstrated a TB1 median of 0.45 (IQR: 0.36–0.95) with an average of 0.618, and a TB2 median of 0.41 (IQR: 0.13–0.66) with an average of 0.493. Average TB1 and TB2 changes in the reversion group were −1.11 ± 0.63 and −1.16 ± 0.42, respectively. In contrast, conversion cases demonstrated average increases of only 0.38 ± 0.17 and 0.37 ± 0.15, respectively.

**Fig 2:**
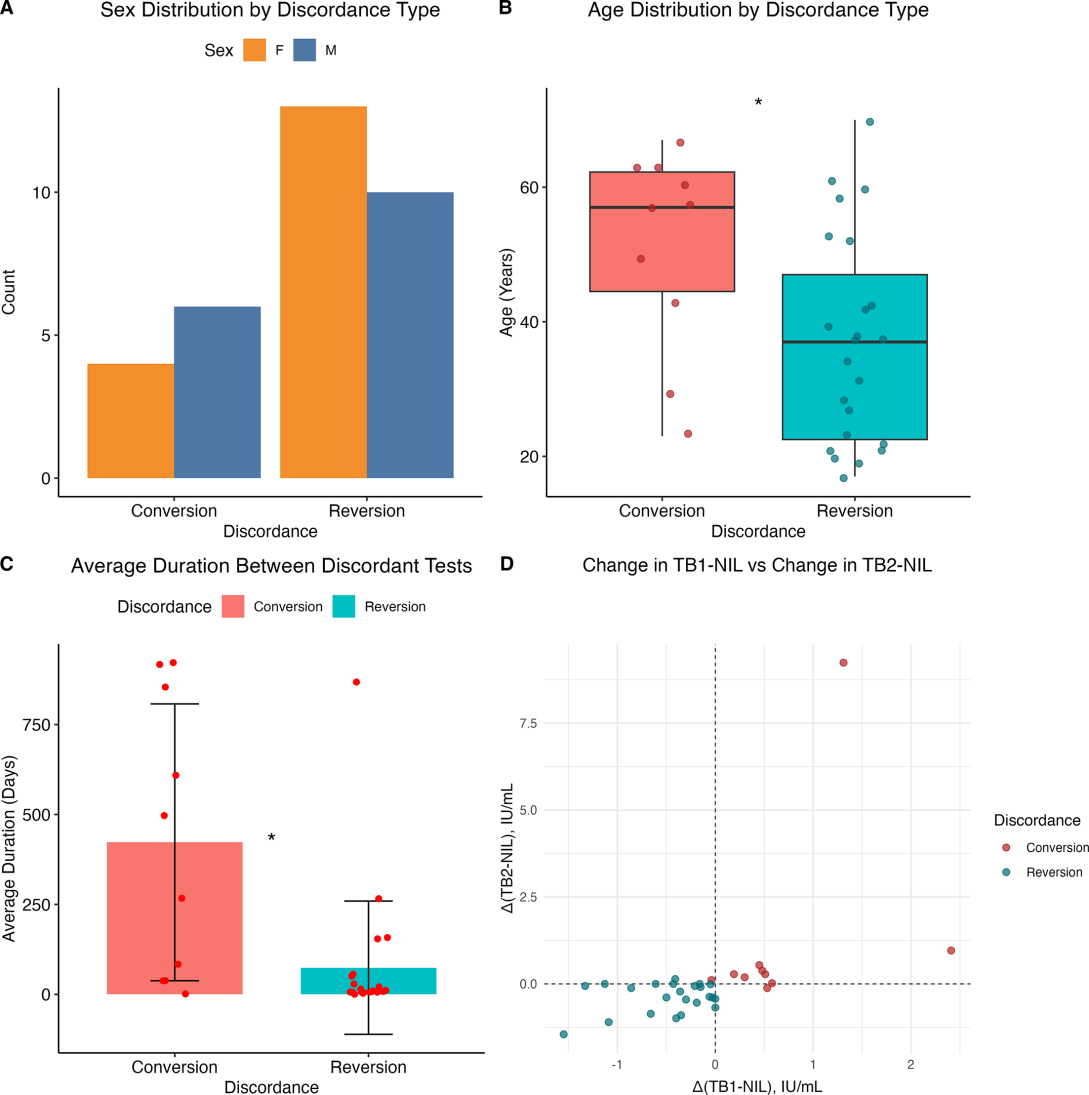
Age and duration between serial testing correlate with QFTTB discordance. (**A**) Sex distribution by discordance group (conversion vs reversion), presented as a bar plot. (**B**) Age distribution by discordance group, shown as boxplots. Boxes represent the IQR, horizontal lines denote the median, and whiskers extend to 1.5 × IQR (Welch’s *t*-test *P =* 0.025*)*. (**C**) Mean time in days between serial QFTTB tests by discordance group, shown as a bar plot with SD error bars (*P* = 0.02574). (**D**) Scatterplot displaying changes in TB1-NIL and TB2-NIL IFN-γ responses among individuals with discordant QFTTB results. Statistical comparisons were performed using independent Welch’s *t*-tests. Asterisks denote levels of statistical significance: *P* < 0.05 (*)

There was no significant association between sex and conversion measured by Fisher’s exact test (*P* = 0.4646; odds ratio = 0.5234; 95% CI: 0.0837–2.9271) (Fig. 2A). Conversion cases were significantly older than reverters (mean age 51.1 ± 15.0 vs 37.0 ± 15.6 years; *t*(17.79) = 2.44, *P* = 0.025; 95% CI: 1.95–26.16) (Fig. 2B). Significantly more time elapsed between discordant results for conversions (415.1 days [SD ± 394] vs 91.2 days [SD ± 196]) (Welch’s test: *t*(10.927) = 2.5794, *P* = 0.02574) (Fig. 2C).

Although converters and reverters are defined by opposing shifts in IFN-γ concentration, the magnitude and distribution of these changes distinguished discordant groups (Fig. 2D). Converters exhibited a wider range of increases in TB1 (median: +0.495 IU/mL, range: –0.04 to +2.41) and TB2 (median: +0.28 IU/mL, range: –0.12 to +9.24), with statistical outliers identified at 2.41 IU/mL (TB1) and 9.24 IU/mL (TB2) based on a *z*-score threshold >2.5. In contrast, reverters demonstrated more uniform decreases in TB1 (median: –0.36 IU/mL, range: –1.55 to 0.00) and TB2 (median: –0.37 IU/mL, range: –1.45 to +0.14), with over half demonstrating TB2 decreases greater than 0.6 IU/mL.

### Impact of clinical factors on discordant results

**Fig 3:**
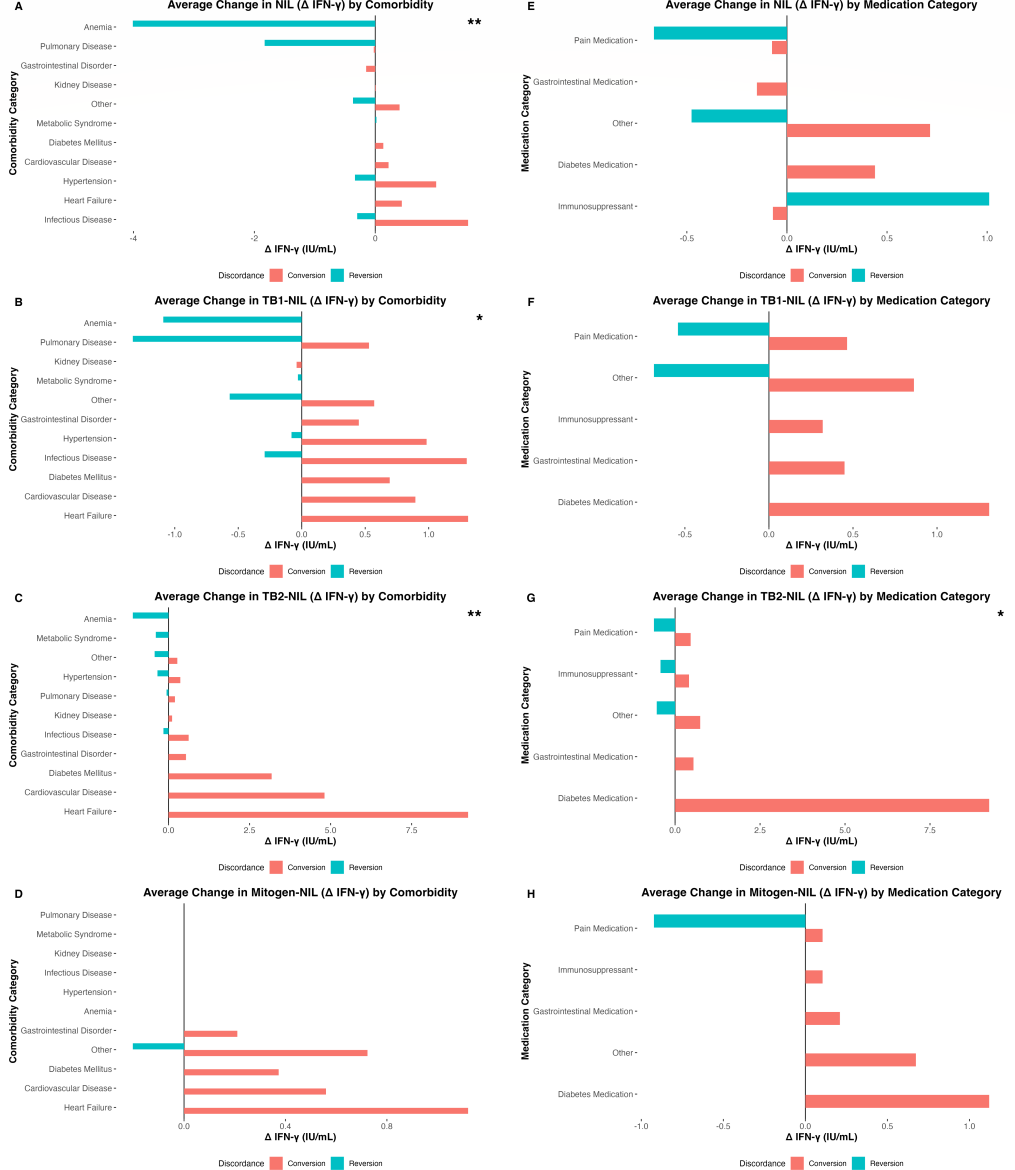
Clinical comorbidities and medications correlate with change in quantitative QFTTB result. Bar plots showing the average change in NIL (**A**), TB1-NIL (**B**), TB2-NIL (**C**), and Mitogen-NIL (**D**) stratified by comorbidity and discordance group (conversion vs reversion). Bar plots showing the average change in NIL (**E**), TB1-NIL (**F**), TB2-NIL (**G**), and Mitogen-NIL (**H**) stratified by medication category and discordance group. Statistical comparisons were performed using a two-way ANOVA. Asterisks denote levels of statistical significance: *P* < 0.05 (*), *P <* 0.01 (**)*.*

We next examined the relationship between comorbidity and changes in NIL, TB1, TB2, and mitogen responses by discordance group (Fig. 3A through D). Comorbidity was significantly associated with changes in NIL (Fig. 3A), TB1 (Fig. 3B), and most notably TB2 (*F*(3, 8) = 275.64, *P* = 1.75 × 10⁻⁷) (Fig. 3C), but not discordance (*F*(3, 8) = 4.90, *P* = 0.058). Comorbidities were not significantly associated with mitogen responses (*F*(3, 8) = 0.025, *P* = 0.994) (Fig. 3D), indicating preserved T-cell function across groups.

Average positive changes in IFN-γ concentration in the NIL condition were highest in individuals with hypertension or infectious diseases, while negative changes correlated with mild anemia or pulmonary disease (Fig. 3A). For TB1, the largest positive changes were observed in individuals with diabetes mellitus or cardiovascular disease, while the largest negative changes were seen in those with anemia or pulmonary disease (Fig. 3B). TB2 responses showed the greatest positive changes in conversion subjects with cardiovascular disease or diabetes mellitus, and the largest negative changes in reverters with pulmonary disease (Fig. 3C). Mitogen responses were similar across comorbidity groups, with nominal increases in converters with comorbidities (Fig. 3D). We also assessed the effects of medication on QFTTB results (Fig. 3E through H). No significant associations were observed between medication use and changes in NIL (Fig. 3E), TB1 (Fig. 3F), or mitogen responses (Fig. 3H). However, TB2-positive changes were significantly associated with medication class (*F*(1, 3) = 26.71, *P* = 0.014), with highest positive changes among converters taking diabetes medications, such as metformin (Fig. 3G).

We observed differences in comorbidity burden between the discordant groups. Among the 23 reversion cases, only 5 (22%) had a serious chronic condition (e.g., HIV and end-stage renal disease), while 18 (78%) had no comorbidities or mild disease (e.g., anemia, dyslipidemia, obesity, or anxiety). In contrast, 8 out of 10 converters (80%) had at least one significant chronic disease (e.g., HIV, diabetes, or cardiovascular disease).

## DISCUSSION

Our single-center study provides important insights into the performance of QFTTB testing in a low-incidence setting for *Mtb* transmission. We found that 16.2% of subjects undergoing serial QFTTB screening demonstrated discordant results. Five percent of subjects with initial positive testing underwent confirmatory testing and demonstrated discordant negative results. Our analyses revealed that reverters were significantly younger and underwent retesting over shorter intervals. Accordingly, converters had more chronic comorbidities and medication use. We conclude that discordant QFTTB results are common and confound accurate assessment of TB risk. As these results may prompt consideration for LTBI treatment, careful interpretation is essential. While 3-month weekly isoniazid-rifapentine or 4-month daily rifampin regimens that are currently first-line therapy for LTBI (23) are generally well-tolerated, they may introduce significant side effects including gastrointestinal intolerance, hepatitis, drug-induced hypersensitivity, and gut dysbiosis (24, 25). As such, interpretation of QFTTB testing results may warrant referral to an infectious diseases specialist to stratify risk and evaluate the appropriateness of LTBI treatment. Frequent repeat testing within short intervals largely contributed to discordant results. Mean testing intervals were shorter among reverters (91 days) than converters (415 days), suggesting that short retesting intervals influence result variability. This reinforces current Centers for Disease Control and Prevention (CDC) recommendations to confirm unexpected positives in low pre-test probability settings before treatment consideration.

A notable finding in our study was the significant age difference between individuals in the conversion and reversion groups with a mean age of 51.1 versus 37.0 years at most recent testing (*P* = 0.0253). This age difference does not appear to be driven by the duration between serial testing (415.1 days [SD ± 394] vs 91.2 days), which was on average less than 2 years of follow-up per case. Most reverters had no significant clinical comorbidities. Younger individuals underwent repeat QFTTB testing over shorter intervals, often in occupational or routine screening contexts, where 5.28% of initially positive results reverted to negative upon retesting. This shorter testing interval among reverters likely represents reflex retesting triggered by low pre-test probability or occupational screening guidelines, where confirmation is recommended. Subjects undergoing TB testing despite low pre-test probability of infection are at increased risk for false-positive tests (26). We believe this is the most likely explanation for the inverse association between reversion and patient age. More than half of the reverters demonstrated TB2 declines greater than 0.6 IU/mL. TB2 antigens include shorter peptide pools designed to trigger both CD8^+^ and CD4^+^ T-cell responses, and both immune lineages are thought to play protective roles against active TB disease (6, 8, 13). We consider that these discordant results may represent expected biological variation in the *Mtb*-specific T-cell response over time rather than analytic error (27, 28). These fluctuations also fall within the reproducibility range previously reported (27), where within-subject IGRA variation near the 0.35 IU/mL threshold can exceed ±0.5 IU/mL and may reduce specificity in low-incidence settings. Our results underscore the importance of existing CDC guidelines to repeat QFTTB testing if the probability of *Mtb* exposure and LTBI are low prior to considering treatment (29).

In contrast, converters had longer intervals between testing that were significantly associated with chronic comorbidities and medication use, namely metformin that correlated with highest change in TB2 values. This suggests that chronic illness and medication use may influence QFTTB responses. Additionally, chronic diseases may be associated with persistent low-grade inflammation, potentially augmenting immune responses and subsequent QFTTB immunoreactivity (30, 31). Older subjects also experience increased cumulative risk of *Mtb* exposure over time, including frequent contact with the healthcare system or foreign travel, that represent potential confounding variables not captured in our study. As of 2024, Suffolk County, New York, reported an active TB case rate of 3.4 per 100,000 annually (5.4 per 100,000 in New York State), making local transmission unlikely (21). In low-incidence populations, the manufacturer’s 0.35 IU/mL cutoff may misclassify borderline results. Small analytic or biologic fluctuations around this threshold can alter classification that may not reflect biologically significant *Mtb* exposure. Prior work supports the use of a “borderline zone” between 0.2 and 0.7 IU/mL or population-specific calibration to improve interpretive accuracy (27). Nonetheless, this must be considered in compliance with regulatory requirements for *in vitro* diagnostic assays, which require adherence to prescribed vendor instructions for use, or off-label validation as laboratory-developed assays. Since low pre-test probability inherently lowers positive predictive value, the higher frequency of apparent false positives in this cohort may reflect expected biological variability rather than technical limitations of the assay. Our results underscore the importance of integrating clinical history and epidemiologic risk when ordering or interpreting QFTTB testing in low-incidence settings.

We acknowledge that additional biological variables that may modulate QFTTB reactivity were not measured in this study. Prior studies report that 8–19% of culture-confirmed active TB cases test IGRA-negative, often in the setting of anergy or immunosuppression, which reflects a biological limitation of T-cell–based assays to diagnose active TB disease rather than a technical error (17, 19, 32). In rare cases, false positives may result from immunologic cross-reactivity with non-tuberculous mycobacterial antigens derived from *M. kansasii* or *M. marinum* (33). Diurnal fluctuation, transient inflammation, recent infections, or vaccinations may also modulate immune responses and QFTTB reliability (5, 28).

Operational variables should also be considered, such as sample handling procedures and quality control, which can contribute to discordance but could not be measured in this study. In the United States, QFTTB testing is required to be carried out in Clinical Laboratory Improvement Amendments (CLIA'88) compliant laboratories that adhere to regulations from bodies such as the College of American Pathologists. This requires adherence to operational temperature ranges and standard operating procedures (SOPs). Deviations from SOPs are flagged in the EMR, and the results are withheld if the criteria are not met. Thus, the results reported pass rigorous quality assurance protocols prior to release. However, operational and pre-analytical variables, including delays in sample delivery to lab and temperature instability during transport or storage, may impact test reproducibility (4, 19, 28, 34). As samples must be processed within 12 hours of collection (35), variability in time to process may contribute to assay fluctuation (36, 37). There are also potential operator-dependent variables in the application of the test itself, including initiation of the procedure, improper tube mixing, and variable incubation period prior to quantitation (28, 37). Discordance may be influenced by any of these operational variables that could not be captured in the study.

We also recognize the limitation of this being a single-center retrospective analysis of QFTTB testing in a region with low TB incidence that did not evaluate alternative IGRA platforms. Testing indications in this cohort—including employee screening, pre-transplant evaluation, and obstetric assessments—represent heterogeneous populations with variable pre-test probabilities and immune backgrounds. This diversity likely contributed to the observed variability in IFN-γ responses. Thus, we expect that our results will be most relevant to other low-incidence clinical settings in which individuals undergo serial QFTTB testing. However, we also believe that our findings may apply to high-incidence settings where serial QFTTB is being used as a surrogate endpoint for incident *Mtb* infection in TB vaccine clinical trials (10, 38, 39). QFTTB values in our cohort clustered between 0.2 and 0.7 IU/mL, the range where analytic and biologic variability are most frequent. As such, the 69.7% reversion rate observed in our study may be consistent with transient antigen responses with spontaneous reversions (40, 41) or fluctuations in immune responsiveness near the cutoff that may not be *Mtb*-specific (27, 42). Discordant serial testing that does not accurately reflect *Mtb*-specific responses can confound both clinical decision-making and interpretation of clinical trial results.

In sum, our results demonstrate significant variability in QFTTB results during serial testing that should raise caution when interpreting positive results and considering LTBI therapy initiation. Coordination with local clinical laboratories, confirmatory testing, and Infectious Diseases specialist consultation may be helpful in assessing these cases. Ongoing work seeks to define the immunologic mechanisms underlying hypothesized non-specific reactions to *Mtb* peptide pools that may be driving QFTTB discordance. This includes examining the relative contribution of alternative IFN-γ-secreting immune subsets, such as natural killer cells and innate-like T cells, relative to conventional, *Mtb* peptide-specific CD4^+^ and CD8^+^ T cells that the assay is designed to target (43–45).

## SUPPLEMENTAL MATERIAL

Figure S1 spectrum.02822-25-s0001.docx Correlates of QFTTB results.

ASM does not own the copyrights to Supplemental Material that may be linked to, or accessed through, an article. The authors have granted ASM a non-exclusive, world-wide license to publish the Supplemental Material files. Please contact the corresponding author directly for reuse.

## References

[1] Tuberculosis (TB). Available from: https://www.who.int/news-room/fact-sheets/detail/tuberculosis. Retrieved 16 Mar 2025.

[2] Sterling TR, Njie G, Zenner D, Cohn DL, Reves R, Ahmed A, Menzies D, Horsburgh CR Jr, Crane CM, Burgos M, LoBue P, Winston CA, Belknap R. 2020. Guidelines for the treatment of latent tuberculosis infection: recommendations from the National Tuberculosis Controllers Association and CDC, 2020. MMWR Recomm Rep 69:1–11. doi:10.15585/mmwr.rr6901a1 PMC7041302 32053584

[3] Lewinsohn DM, Leonard MK, LoBue PA, Cohn DL, Daley CL, Desmond E, Keane J, Lewinsohn DA, Loeffler AM, Mazurek GH, O’Brien RJ, Pai M, Richeldi L, Salfinger M, Shinnick TM, Sterling TR, Warshauer DW, Woods GL. 2017. Official American Thoracic Society/Infectious Diseases Society of America/Centers for Disease Control and Prevention clinical practice guidelines: diagnosis of tuberculosis in adults and children. Available from: https://www.idsociety.org/practice-guideline/diagnosis-of-tb-in-adults-and-children/. Retrieved 05 Jun 2025. 10.1093/cid/ciw778 PMC5504475 28052967

[4] CDC. 2024. Clinical testing guidance for tuberculosis: interferon gamma release assay. Tuberculosis (TB). Available from: https://www.cdc.gov/tb/hcp/testing-diagnosis/interferon-gamma-release-assay.html. Retrieved 06 Apr 2025.

[5] Pai M, Denkinger CM, Kik SV, Rangaka MX, Zwerling A, Oxlade O, Metcalfe JZ, Cattamanchi A, Dowdy DW, Dheda K, Banaei N. 2014. Gamma interferon release assays for detection of Mycobacterium tuberculosis infection. Clin Microbiol Rev 27:3–20. doi:10.1128/CMR.00034-13 24396134 PMC3910908

[6] Tsuyuzaki M, Igari H, Okada N, Suzuki K. 2020. Role of CD8 T-cell in immune response to tuberculosis-specific antigen in QuantiFERON-TB Gold Plus. J Infect Chemother 26:570–574. doi:10.1016/j.jiac.2020.01.011 32067903

[7] CD8 T cell technology. TB testing. QuantiFERON. Available from: http://www.qiagen.com/us/tb-testing/what-is-quantiferon/cd8-technology. Retrieved 06 Apr 2025.

[8] Qiagen. 2023. QuantiFERON-TB Gold Plus ELISA package insert. Available from: https://www.quantiferon.com/wp-content/uploads/2017/04/English_QFTPlus_ELISA_R04_022016.pdf. Retrieved 03 Jun 2025.

[9] 2021. WHO operational handbook on tuberculosis. Module 2: screening - systematic screening for tuberculosis disease. 1st ed. World Health Organization. 33822560

[10] Nemes E, Geldenhuys H, Rozot V, Rutkowski KT, Ratangee F, Bilek N, Mabwe S, Makhethe L, Erasmus M, Toefy A, et al.. 2018. Prevention of infection with Mycobacterium tuberculosis by H4:IC31 vaccination or BCG revaccination in adolescents. N Engl J Med 379:138–149. doi:10.1056/nejmoa1714021 29996082 PMC5937161

[11] Pollock NR, Campos-Neto A, Kashino S, Napolitano D, Behar SM, Shin D, Sloutsky A, Joshi S, Guillet J, Wong M, Nardell E. 2008. Discordant QuantiFERON-TB Gold test results among us healthcare workers with increased risk of latent tuberculosis infection: a problem or solution? Infect Control Hosp Epidemiol 29:878–886. doi:10.1086/590262 18713053 PMC3578293

[12] Stout JE, Belknap R, Wu Y-J, Ho CS, Tuberculosis Epidemiologic Studies Consortium. 2018. Paradox of serial interferon-gamma release assays: variability width more important than specificity size. Int J Tuberc Lung Dis 22:518–523. doi:10.5588/ijtld.17.0650 29663956 PMC9132738

[13] Goletti D, Delogu G, Matteelli A, Migliori GB. 2022. The role of IGRA in the diagnosis of tuberculosis infection, differentiating from active tuberculosis, and decision making for initiating treatment or preventive therapy of tuberculosis infection. Int J Infect Dis 124:S12–S19. doi:10.1016/j.ijid.2022.02.047 35257904

[14] Winglee K, Hill AN, Belknap R, Stout JE, Ayers TL. 2022. Variability of interferon-γ release assays in people at high risk of tuberculosis infection or progression to tuberculosis disease living in the United States. Clin Microbiol Infect 28:1023. doi:10.1016/j.cmi.2022.02.020 PMC10065409 35183749

[15] de Visser V, Sotgiu G, Lange C, Aabye MG, Bakker M, Bartalesi F, Brat K, Chee CBE, Dheda K, Dominguez J, et al.. 2015. False-negative interferon-γ release assay results in active tuberculosis: a TBNET study. Eur Respir J 45:279–283. doi:10.1183/09031936.00120214 25359336

[16] Price C, Nguyen AD. 2025. Latent tuberculosis. StatPearls. 38261712

[17] Yamasue M, Komiya K, Usagawa Y, Umeki K, Nureki S-I, Ando M, Hiramatsu K, Nagai H, Kadota J-I. 2020. Factors associated with false negative interferon-γ release assay results in patients with tuberculosis: a systematic review with meta-analysis. Sci Rep 10:1607. doi:10.1038/s41598-020-58459-9 32005930 PMC6994686

[18] Adams S, Ehrlich R, Baatjies R, Dendukuri N, Wang Z, Dheda K. 2019. Predictors of discordant latent tuberculosis infection test results amongst South African health care workers. BMC Infect Dis 19:131. doi:10.1186/s12879-019-3745-5 30736743 PMC6368796

[19] Santos JA, Duarte R, Nunes C. 2020. Host factors associated to false negative and indeterminate results in an interferon‐γ release assay in patients with active tuberculosis. Pulmonology 26:353–362. doi:10.1016/j.pulmoe.2019.11.001 31843341

[20] Zhang Y, Zhou G, Shi W, Shi W, Hu M, Kong D, Long R, He J, Chen N. 2023. Comparing the diagnostic performance of QuantiFERON-TB Gold Plus with QFT-GIT, T-SPOT.TB and TST: a systematic review and meta-analysis. BMC Infect Dis 23:40. doi:10.1186/s12879-023-08008-2 36670347 PMC9862551

[21] Tuberculosis data and statistics. Available from: https://www.health.ny.gov/statistics/diseases/communicable/tuberculosis/. Retrieved 19 May 2025.

[22] 1.1 TB incidence. Available from: https://www.who.int/teams/global-programme-on-tuberculosis-and-lung-health/tb-reports/global-tuberculosis-report-2024/tb-disease-burden/1-1-tb-incidence. Retrieved 28 Oct 2025.

[23] CDC. 2025. Treatment for latent tuberculosis infection. Tuberculosis (TB). Available from: https://www.cdc.gov/tb/hcp/treatment/latent-tuberculosis-infection.html. Retrieved 19 May 2025.

[24] Singh KP, Carvalho ACC, Centis R, D Ambrosio L, Migliori GB, Mpagama SG, Nguyen BC, Aarnoutse RE, Aleksa A, van Altena R, et al.. 2023. Clinical standards for the management of adverse effects during treatment for TB. Int J Tuberc Lung Dis 27:506–519. doi:10.5588/ijtld.23.0078 37353868 PMC10321364

[25] Wipperman MF, Fitzgerald DW, Juste MAJ, Taur Y, Namasivayam S, Sher A, Bean JM, Bucci V, Glickman MS. 2017. Antibiotic treatment for Tuberculosis induces a profound dysbiosis of the microbiome that persists long after therapy is completed. Sci Rep 7:10767. doi:10.1038/s41598-017-10346-6 28883399 PMC5589918

[26] Shreffler J, Huecker MR. 2025. Diagnostic testing accuracy: sensitivity, specificity, predictive values and likelihood ratios. StatPearls. 32491423

[27] Tagmouti S, Slater M, Benedetti A, Kik SV, Banaei N, Cattamanchi A, Metcalfe J, Dowdy D, van Zyl Smit R, Dendukuri N, Pai M, Denkinger C. 2014. Reproducibility of interferon gamma (IFN-γ) release assays. A systematic review. Ann Am Thorac Soc 11:1267–1276. doi:10.1513/AnnalsATS.201405-188OC 25188809 PMC5469356

[28] Metcalfe JZ, Cattamanchi A, McCulloch CE, Lew JD, Ha NP, Graviss EA. 2013. Test variability of the QuantiFERON-TB Gold in-tube assay in clinical practice. Am J Respir Crit Care Med 187:206–211. doi:10.1164/rccm.201203-0430OC 23103734 PMC3570654

[29] Updated guidelines for using interferon gamma release assays to detect Mycobacterium tuberculosis infection --- United States, 2010. 2010. Available from: https://www.cdc.gov/mmwr/preview/mmwrhtml/rr5905a1.htm. Retrieved 01 Jun 2025. 20577159

[30] Franceschi C, Garagnani P, Parini P, Giuliani C, Santoro A. 2018. Inflammaging: a new immune–metabolic viewpoint for age-related diseases. Nat Rev Endocrinol 14:576–590. doi:10.1038/s41574-018-0059-4 30046148

[31] Furman D, Campisi J, Verdin E, Carrera-Bastos P, Targ S, Franceschi C, Ferrucci L, Gilroy DW, Fasano A, Miller GW, Miller AH, Mantovani A, Weyand CM, Barzilai N, Goronzy JJ, Rando TA, Effros RB, Lucia A, Kleinstreuer N, Slavich GM. 2019. Chronic inflammation in the etiology of disease across the life span. Nat Med 25:1822–1832. doi:10.1038/s41591-019-0675-0 31806905 PMC7147972

[32] Sester M, Sotgiu G, Lange C, Giehl C, Girardi E, Migliori GB, Bossink A, Dheda K, Diel R, Dominguez J, Lipman M, Nemeth J, Ravn P, Winkler S, Huitric E, Sandgren A, Manissero D. 2011. Interferon-γ release assays for the diagnosis of active tuberculosis: a systematic review and meta-analysis. Eur Respir J 37:100–111. doi:10.1183/09031936.00114810 20847080

[33] Augustynowicz-Kopeć E, Siemion-Szcześniak I, Zabost A, Wyrostkiewicz D, Filipczak D, Oniszh K, Gawryluk D, Radzikowska E, Korzybski D, Szturmowicz M. 2019. Interferon gamma release assays in patients with respiratory isolates of non-tuberculous mycobacteria – a preliminary study. Pol J Microbiol 68:15–19. doi:10.21307/pjm-2019-002 31050249 PMC7256814

[34] Jarvis J, Gao Y, de Graaf H, Hughes S, Allan RN, Williams A, Marshall B, Elkington P, Faust SN, Tebruegge M. 2015. Environmental temperature impacts on the performance of QuantiFERON-TB Gold in-tube assays. J Infect 71:276–280. doi:10.1016/j.jinf.2015.04.004 25869537

[35] How to Test with QuantiFERON TB. QFT-Plus. QIAGEN. Available from: http://www.qiagen.com/us/tb-testing/what-is-quantiferon/how-does-qft-work. Retrieved 01 Jun 2025.

[36] Doberne D, Gaur RL, Banaei N. 2011. Preanalytical delay reduces sensitivity of QuantiFERON-TB gold in-tube assay for detection of latent tuberculosis infection. J Clin Microbiol 49:3061–3064. doi:10.1128/JCM.01136-11 21697332 PMC3147723

[37] Faria N, Reis R. 2022. Screening for TB infection: the operator´s impact. Int J Tuberc Lung Dis 26:857–861. doi:10.5588/ijtld.22.0052 35996296

[38] Lu LL, Smith MT, Yu KKQ, Luedemann C, Suscovich TJ, Grace PS, Cain A, Yu WH, McKitrick TR, Lauffenburger D, Cummings RD, Mayanja-Kizza H, Hawn TR, Boom WH, Stein CM, Fortune SM, Seshadri C, Alter G. 2019. IFN-γ-independent immune markers of Mycobacterium tuberculosis exposure. Nat Med 25:977–987. doi:10.1038/s41591-019-0441-3 31110348 PMC6559862

[39] Van Dis E, Fox DM, Morrison HM, Fines DM, Babirye JP, McCann LH, Rawal S, Cox JS, Stanley SA. 2022. IFN-γ-independent control of M. tuberculosis requires CD4 T cell-derived GM-CSF and activation of HIF-1α. PLoS Pathog 18:e1010721. doi:10.1371/journal.ppat.1010721 35877763 PMC9352196

[40] van Zyl-Smit RN, Pai M, Peprah K, Meldau R, Kieck J, Juritz J, Badri M, Zumla A, Sechi LA, Bateman ED, Dheda K. 2009. Within-subject variability and boosting of T-cell interferon-gamma responses after tuberculin skin testing. Am J Respir Crit Care Med 180:49–58. doi:10.1164/rccm.200811-1704OC 19342414

[41] Ringshausen FC, Schablon A, Nienhaus A. 2012. Interferon-gamma release assays for the tuberculosis serial testing of health care workers: a systematic review. J Occup Med Toxicol 7:6. doi:10.1186/1745-6673-7-6 22537915 PMC3377540

[42] Banaei N, Gaur RL, Pai M. 2016. Interferon gamma release assays for latent tuberculosis: what are the sources of variability? J Clin Microbiol 54:845–850. doi:10.1128/JCM.02803-15 26763969 PMC4809912

[43] Mehanna N, Pradhan A, Kaur R, Kontopoulos T, Rosati B, Carlson D, Cheung N-K, Xu H, Bean J, Hsu KC, Le Luduec J-B, Vorkas CK. 2025. CD8α marks a Mycobacterium tuberculosis-reactive human NK cell population with high activation potential. Sci Rep 15:15095. doi:10.1038/s41598-025-98367-4 40301594 PMC12041513

[44] Cross DL, Layton ED, Yu KK, Smith MT, Aguilar MS, Li S, Wilcox EC, Chapuis AG, Mayanja-Kizza H, Stein CM, Boom WH, Hawn TR, Bradley P, Newell EW, Seshadri C. 2024. MR1-restricted T cell clonotypes are associated with “resistance” to Mycobacterium tuberculosis infection. JCI Insight 9:e166505. doi:10.1172/jci.insight.166505 38716731 PMC11141901

[45] Vorkas CK, Wipperman MF, Li K, Bean J, Bhattarai SK, Adamow M, Wong P, Aubé J, Juste MAJ, Bucci V, Fitzgerald DW, Glickman MS. 2018. Mucosal-associated invariant and γδ T cell subsets respond to initial Mycobacterium tuberculosis infection. JCI Insight 3:121899. doi:10.1172/jci.insight.121899 30282828 PMC6237486

