# Supplementary material for: Demographic and clinical correlates of discordant QuantiFERON TB Gold tuberculosis screening results in a low-incidence setting
Source: Microbiol Spectr. 2026 Feb 9;14(3):e02822-25. doi: 10.1128/spectrum.02822-25 (PMC12955443; doi:10.1128/spectrum.02822-25)
Supplement: Figure S1 — Correlates of QFTTB results. [file spectrum.02822-25-s0001.docx]

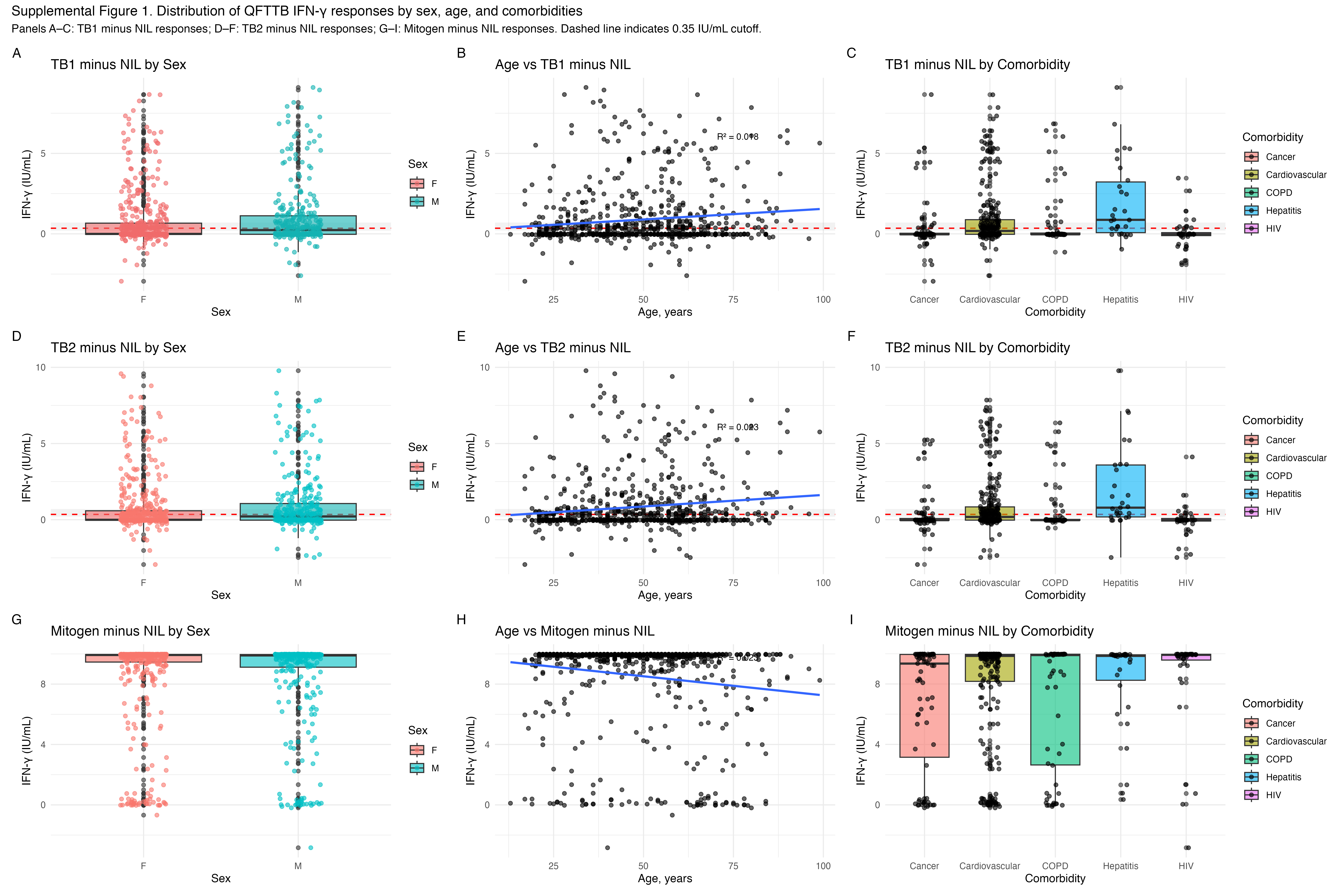


**Supplemental Figure 1.** Distribution of QFTTB IFN-γ responses by sex, age, and medical comorbidities. TB1-NIL IFN-γ values stratified by sex (**A**), plotted against age with linear regression (**B**), and shown across comorbidity categories (**C**). TB2-NIL IFN-γ values stratified by sex (**D**), plotted against age with linear regression (**E**), and shown across comorbidity categories (**F**). Mitogen-NIL IFN-γ values stratified by sex (**G**), plotted against age with linear regression (**H**), and shown across comorbidity categories (**I**). Each panel displays mean IFN-γ concentrations (IU/mL) as measured by QFTTB component assays. Statistical comparisons were performed using linear regression or ANOVA as appropriate.
